# Comparison of the Orbscan II topographer and the iTrace aberrometer for the measurements of keratometry and corneal diameter in myopic patients

**DOI:** 10.1186/s12886-016-0210-8

**Published:** 2016-03-31

**Authors:** Yao Chen, Xiaobo Xia

**Affiliations:** Department of Ophthalmology, Xiangya Hospital, Central South University, Changsha, Hunan China

**Keywords:** Keratometry, White-to-white distance, Orbscan II, iTrace

## Abstract

**Background:**

The purpose of this study was to compare corneal power and horizontal corneal diameter (white-to-white [WTW] distance) readings obtained by the Orbscan II topographer and the iTrace aberrometer.

**Methods:**

Keratometry readings in the flat (Kf) and steep (Ks) meridians and WTW distance were measured with the Orbscan II and iTrace systems in 100 myopic patients. Statistical evaluation was performed using the paired *t* test, Pearson correlation, and Bland-Altman analysis for comparison of measurement techniques.

**Results:**

The mean keratometry values with the Orbscan II and iTrace were 43.16 ± 1.44 and 42.64 ± 1.43 diopter (D), respectively (*P* < 0.0001). The mean WTW distance measurements with the Orbscan II and iTrace were 11.57 ± 0.34 and 11.33 ± 0.36 mm, respectively (*P* < 0.0001). For the measurement of corneal power, the 95 % limits of agreement (LoA) between the Orbscan II and iTrace were − 0.21 to 1.21 D for the flat meridian and − 0.15 to 1.25 D for the steep meridian. For the measurement of WTW distance, the range of the 95 % LoA between the two devices was 0.47 mm.

**Conclusions:**

For some clinical applications, the keratometry and WTW distance measurements obtained by the Orbscan II topographer and the iTrace aberrometer differed greatly and therefore were not interchangeable.

**Trial registration:**

Clinical trials number: ChiCTR-OCS-14005077 (August 2nd, 2014).

## Background

Measurements of corneal power and WTW distance are important prior to either cataract or refractive surgery. Accurate intraocular lens (IOL) power calculations, contact lens fitting, and monitoring postoperative cornea are major clinical applications of these parameters [1, 2]. The IOL power is usually calculated using standard IOL calculation formulas, which are based on the value of corneal power [3]. Precise keratometry measurements are particularly crucial in determining the correct IOL power for patients who had previously undergone corneal refractive surgery [4, 5]. With the wider use of phakic IOLs, accurate determination of the WTW distance has gained increased attention in sizing posterior chamber phakic IOLs [6, 7] and estimating the postoperative vault height in eyes with implantable collamer lenses (ICL; STAAR Surgical AG, Nidau, Switzerland) [8, 9].

Until recently, the Orbscan II topography system (Bausch & Lomb, Orbtek Inc., UT, USA) has been widely applied for measuring the corneal power and WTW distance and is considered accurate and reproducible [10–14]. Currently, corneal wavefront analysis has gained increased importance in-line with the development of wavefront sensing technology [15, 16]. Clinical wavefront aberrometers allow an objective measurement of optical aberrations other than sphere and cylinder, such as spherical aberration, coma, trefoil, and other higher-order optical aberrations (HOAs), which present diagnostic and therapeutic applications [17].

The commercially available iTrace system (Tracey Technologies Corp. TX, USA) integrates corneal topography with a ray-tracing aberrometer, yielding information about refractive, wavefront and corneal topographic data of the human optical system [18, 19]. Visser et al. [20] showed that the iTrace device exhibits a high level of repeatability for measuring corneal aberrations. However, the measurements of corneal power and WTW distance is seldom reported. Moreover, it remains unclear whether or not the iTrace aberrometer could be considered as an alternative instrument for IOL power calculations in clinical practice. Therefore, the purpose of this study was to compare the corneal power and WTW distance measurements using the Orbscan II topographer and the iTrace aberrometer.

## Methods

### Subjects

One hundred right eyes of 100 myopic patients (51 male, 49 female, age 20.18 ± 5.12 years, age range, 8 to 39 years old) were included in this study. Each subject underwent full ophthalmic examinations, including vision, subjective refraction, slit-lamp and fundus examinations, and corneal topography by the Orbscan II topographer and the iTrace aberrometer. All subjects had good best corrected distance visual acuity (BCVA) equal to or better than 20/20 to allow for adequate fixation. Exclusion criteria included coexisting ocular diseases or a dry eye, corneal anomalies, contact lens wear within the preceding 2 weeks, any history of ocular surgery or trauma. The ethics committee of Xiangya Hospital approved this study. Adults and parents of juveniles provided the written informed consent in accordance with the Declaration of Helsinki before the measurements were carried out.

### Instruments

The Orbscan II topographer, combined slit-scanning with a Placido disk, has the capacity to directly acquire elevation and curvature data of both anterior and posterior corneal surfaces [11]. The Placido disk and 40 slits are sequentially projected on the cornea, then the anterior and posterior edges of the slits are captured and analyzed, and eventually, elevation and curvature topographic maps are generated. The corneal limbus (the border of the cornea and the sclera) is automatically detected to calculate the WTW distance.

The iTrace aberrometer utilizes a Placido disk format named Vista developed by EyeSys Vision Inc. (Houston, TX, USA) with a laser diode at the wavelength of 655 nm for corneal topography assessment. The iTrace software then defines the ring edges and calculates corneal curvature, corneal wavefront data and detects the WTW distance.

### Measurements

The refraction of each eye was determined with subjective manifest refraction. Three repeated consecutive measurements were performed independently by two experienced operators for both eyes of all subjects using the Orbscan II topographer and the iTrace aberrometer. The sequence of the measurements with the two devices was randomly chosen. In this study, the following parameters were recorded, the keratometry readings in the flat (Kf) and steep (Ks) meridians, and the WTW distance. The mean keratometry value was calculated using the following formula: (Kf + Ks)/2. Only the right eye of each subject was calculated and analyzed. All participants underwent measurements approximately 5 min apart between 8 AM and 12 AM.

For the Orbscan II topographer measurements, the operator adjusted the distance between the corneal apex and the center of the moving slit for correct alignment. Then the subjects were asked to keep their eyes open and not move their eyes once the scan had started. The device detected excessive eye movement and discarded low-quality or incomplete images.

For the iTrace aberrometer measurements, the internal optometer incorporated in the device was used for alignment of the patient’s line of sight with the laser axis. Then the iTrace aberrometer automatically centered onto the pupil and verified focus and alignment, and captured the data. The best scan, defined as all of the reflected Placido rings devoid of missing ring edges, was chosen for the final analysis.

### Statistical analysis

Statistical analysis was performed using SPSS software (version 19.0, SPSS, Inc.) and MedCalc (version 13.0, MedCalc software bvba, Inc.). The Kolmogorov-Smirnov test was used to confirm the normality of all data distribution. Differences between the devices were assessed using the paired *t* test. All tests were two tailed, *P* values less than 0.05 were considered statistically significant. The Pearson correlation coefficient was determined to show the correlation between the two measurements for each subject. The Bland-Altman plots with 95 % limits of agreement (LoA; mean difference of two methods ± 1.96 standard deviation) [21] were calculated to evaluate interdevice agreement and interchangeability.

## Results

For 100 right eyes of 100 myopic patients, the mean spherical equivalent refraction was − 5.33 ± 2.42 diopter (D) (range,−12.50 to − 0.75 D). The mean examination time was 10 min, and the iTrace aberrometer was quicker than the Orbscan II topographer for all measurements. No significant deviation from a normal distribution pattern was observed in the corneal power and WTW distance (Kolmogorov-Smirnov tests, *P* > 0.1).

The corneal power and WTW distance readings assessed with the Orbscan II topographer and the iTrace aberrometer are summarized in Table 1. The Kf, Ks, and WTW distance measurements taken with the Orbscan II topographer were greater in magnitude than those measured by the iTrace aberrometer (*P* < 0.0001 for all pairwise comparisons; paired *t* test). The mean keratometry values obtained with the Orbscan II topographer and the iTrace aberrometer were 43.16 ± 1.44 and 42.64 ± 1.43 D, respectively. A statistically significant difference between the two instruments was noted (*P* < 0.0001, paired *t* test). The mean difference (with 95 % LoA) in the mean keratometry measurements between the two instruments was 0.52 D (range:−0.16 to 1.21 D).

**Table 1:** Comparison of the corneal power and WTW distance values measured by the 2 devices

Parameters	Orbscan II	iTrace	*P* value^a^
Kf (D)	42.56 ± 1.34	42.06 ± 1.34	<0.0001
Ks (D)	43.76 ± 1.58	43.21 ± 1.57	<0.0001
WTW (mm)	11.57 ± 0.34	11.33 ± 0.36	<0.0001

Data are expressed as mean ± SD; ^a^paired *t* test <0.05 considered significant; Kf = flat axis power; Ks = steep axis power, D = diopter

**Fig. 1:**
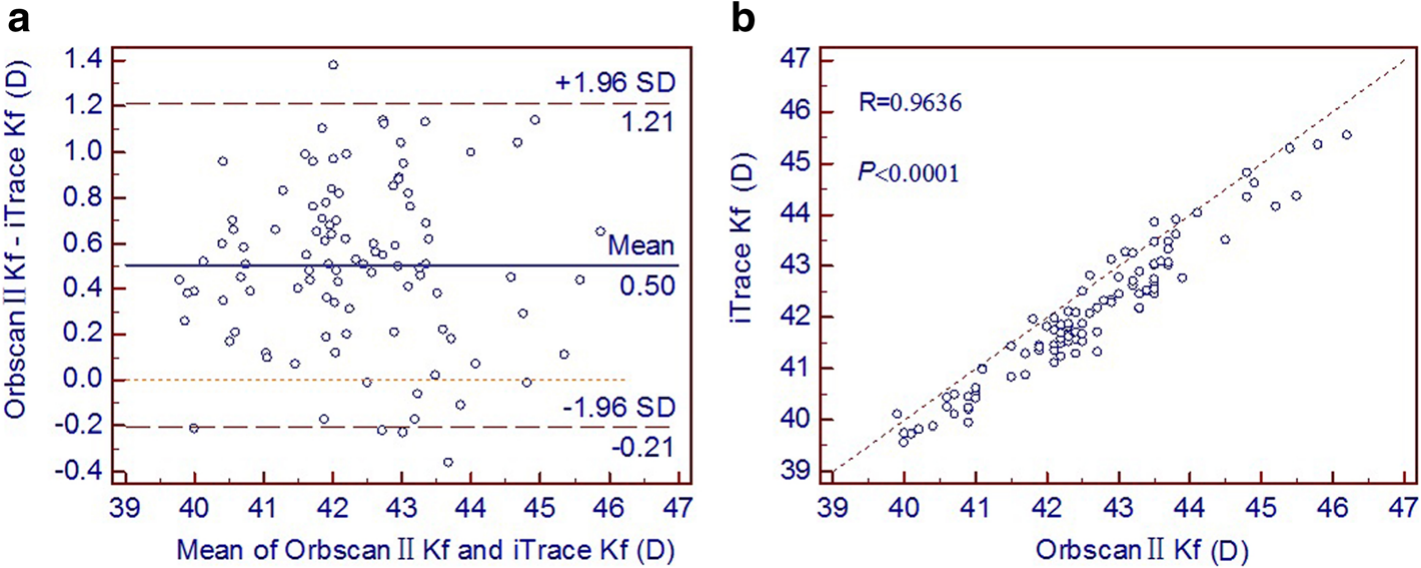
Kf measurements by the Orbsan II versus the iTrace. **a** Bland-Altman plot showing the mean difference and the limits of agreement. **b** Scatter diagram and Pearson correlation analysis

**Fig. 2:**
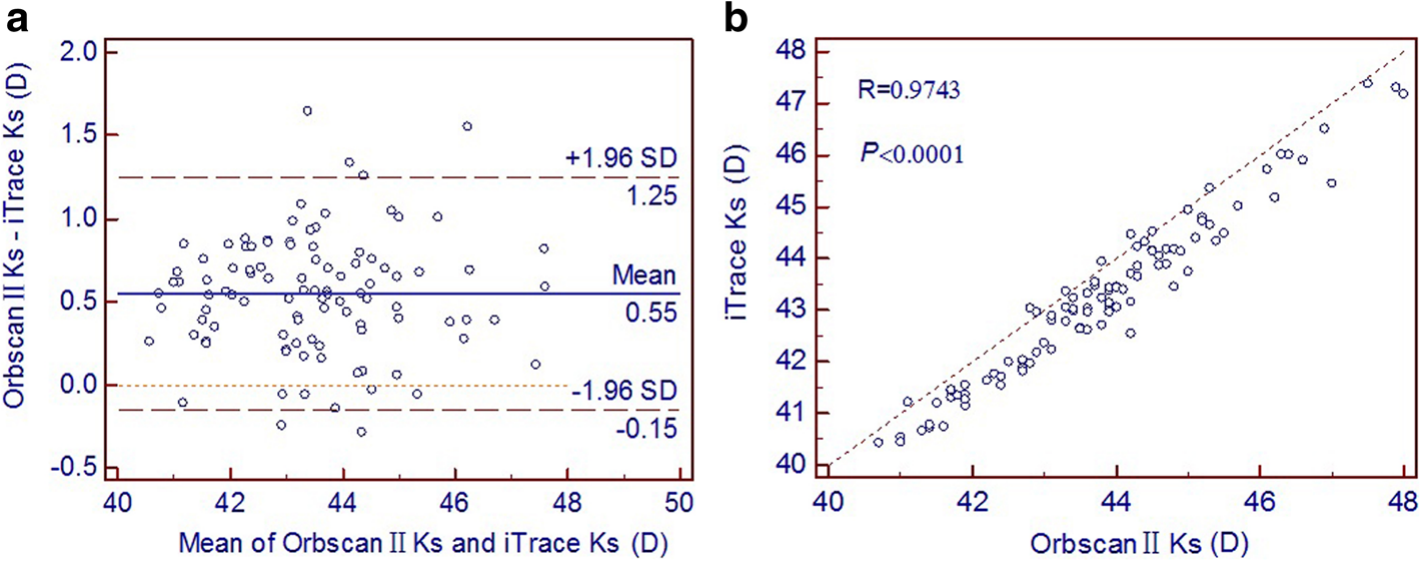
Ks measurements by the Orbsan II versus the iTrace. **a** Bland-Altman plot. **b** Scatter diagram and Pearson correlation analysis

**Fig. 3:**
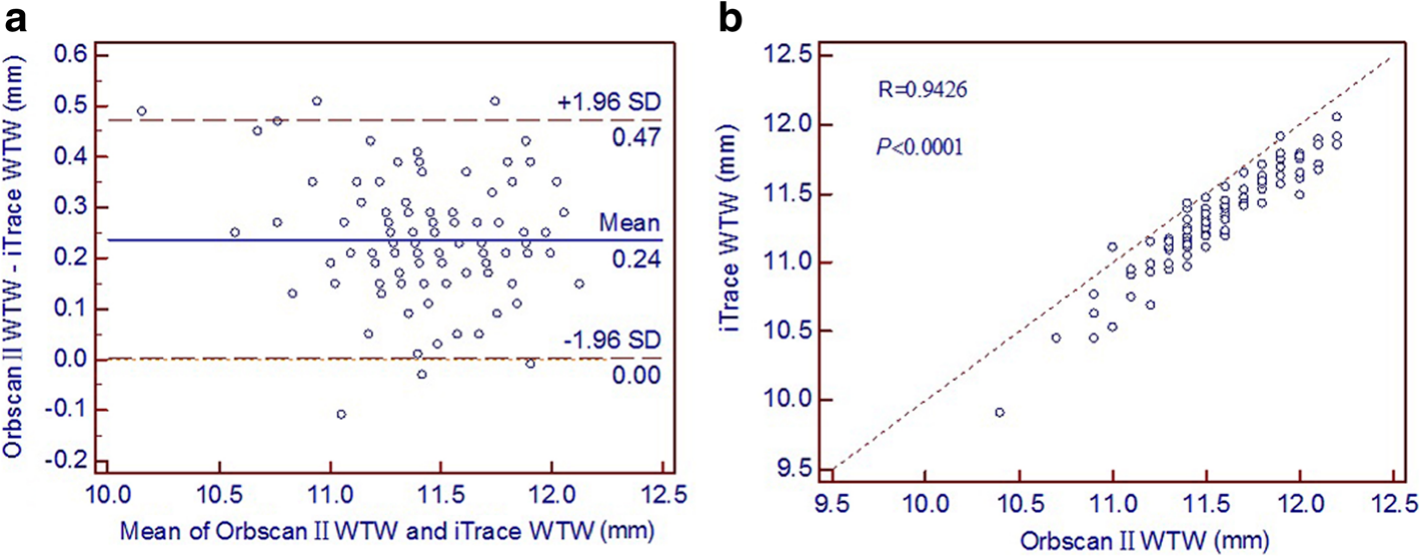
The WTW distance measurements by the Orbsan II versus the iTrace. **a** Bland-Altman plot. **b** Scatter diagram and Pearson correlation analysis

Pearson correlation coefficients and Bland-Altman plots for the measurements of corneal power and WTW distance are shown in Figs. 1, 2 and 3. Results with the Orbscan II topographer and the iTrace aberrometer correlated closely, with Pearson correlation coefficients ranging from 0.9426 to 0.9743. The Bland-Altman plots revealed a fixed bias towards the Orbscan II topographer for the measurements of Kf, Ks and WTW distance. Mean differences (with 95 % LoA) between the Orbscan II topographer and the iTrace aberrometer were 0.50 D (range:−0.21 to 1.21 D) for Kf and 0.55 D (range:−0.15 to 1.25 D) for Ks measurements. The mean difference (with 95 % LoA) in the WTW distance measurements between the two devices was 0.24 mm (range: 0.00 to 0.47 mm).

Based on the median age (19 years), the subjects were divided into age <19 years old (less than 19 years old; mean age, 15.95 ± 2.92; *n* = 40) and age ≥19 years old groups (19 years or older; mean age, 23.00 ± 4.27; *n* = 60). We evaluated the effects of age on the corneal power and WTW distance measured by the Orbscan II topographer and the iTrace aberrometer. The Orbscan II topographer generated larger values when compared with those of the iTrace aberrometer for both age <19 years old and age ≥19 years old groups (*P* < 0.0001 for all pairwise comparisons; paired *t* test; Table 2). The 95 % LoA between the Orbscan II and iTrace were almost larger than 1.20 D for Kf and Ks mesurements for both age <19 years old and age ≥19 years old groups. The 95 % LoA for the WTW distance mesurements were equal to or larger than 0.45 mm for both groups.

**Table 2:** Comparison of the corneal power and WTW distance measurements in age <19 years old and age ≥19 years old groups

groups	parameters	Orbscan II	iTrace	*P* Value^a^	95 % LoA
age <19	Kf (D)	42.29 ± 1.38	41.84 ± 1.45	<0.0001	−0.16,1.07
years old	Ks (D)	43.47 ± 1.65	42.95 ± 1.71	<0.0001	−0.08,1.11
(*n* = 40)	WTW (mm)	11.60 ± 0.29	11.37 ± 0.30	<0.0001	0.00,0.45
age ≥19	Kf (D)	42.74 ± 1.30	42.20 ± 1.26	<0.0001	−0.23,1.30
years old	Ks (D)	43.96 ± 1.51	43.39 ± 1.45	<0.0001	−0.19,1.34
(*n* = 60)	WTW (mm)	11.55 ± 0.36	11.30 ± 0.39	<0.0001	0.01,0.48

Data are expressed as mean ± SD; ^a^paired *t* test <0.05 considered significant; Kf = flat axis power; Ks = steep axis power, D = diopter, LoA = limits of agreement

As shown in Table 3, there were statistically significant differences in the Kf, Ks, and WTW distance mesurements between the Orbscan II topographer and the iTrace aberrometer (*P* < 0.0001 for all pairwise comparisons; paired *t* test) for both male and female subjects. The 95 % LoA for the Kf and Ks mesurements between the Orbscan II and iTrace were equal to or larger than 1.29 D for both male and female subjects. As regards the WTW distance mesurements, the 95 % LoA were equal to or larger than 0.47 mm for both male and female subjects.

**Table 3:** Comparison of the corneal power and WTW distance measurements in male and female subjects

gender	parameters	Orbscan II	iTrace	*P* Value^a^	95 % LoA
Male	Kf (D)	42.12 ± 1.39	41.63 ± 1.37	<0.0001	−0.28,1.26
(*n* = 51)	Ks (D)	43.34 ± 1.63	42.79 ± 1.65	<0.0001	−0.17,1.26
	WTW (mm)	11.64 ± 0.32	11.40 ± 0.33	<0.0001	0.01,0.47
Female	Kf (D)	43.02 ± 1.14	42.51 ± 1.16	<0.0001	−0.13,1.16
(*n* = 49)	Ks (D)	44.20 ± 1.41	43.65 ± 1.36	<0.0001	−0.14,1.24
	WTW (mm)	11.49 ± 0.34	11.25 ± 0.37	<0.0001	−0.01,0.48

Data are expressed as mean ± SD; ^a^paired *t* test <0.05 considered significant; Kf = flat axis power; Ks = steep axis power, D = diopter, LoA = limits of agreement

## Discussion

Refractive considerations are integrated in modern cataract surgery as a result of the increased application of advanced IOLs, progresses in surgical techniques, and use of new preoperative biometry instruments [22, 23]. The final refractive outcome following cataract surgery is affected by various factors such as IOL power calculations, selection of the proper IOL formula, and the quality of the IOL. Of these factors, inaccurate computation of IOL power contributes to the prediction deviations of refractive outcome the most [3]. The accuracy of optical IOL power calculation depends on the preoperative biometric measurements of the eye. Norrby et al. [24] demonstrated that inaccurate corneal power is a major cause of error in IOL power calculations. A 1 D error in the corneal power mesurement will induce approximately a 1 D error in the calculation of the IOL power [25]. Thus, precise postoperative refractive outcomes depend on the improvements in biometry and IOL power calculations.

In this study, measurements of the corneal power and WTW distance obtained by the Orbscan II topographer differed significantly from those of the iTrace aberrometer. Despite a strong positive correlation, almost all plots lied by one side towards the Orbscan II topographer along the equality line (the right-hand side of Figs. 1, 2 and 3). As Bland and Altman [21] pointed out, perfect agreement merely exists if all points lie along the equality line, but perfect correlation will occur if points lie along any straight line. They advocated the use of the 95 % LoA as a more accurate method for assessing agreement. A narrower 95 % LoA indicates better agreement between devices. The acceptable range of agreement between two devices partly depends on clinical practice, that is, if the range of the 95 % LoA is small enough to avoid problems with clinical interpretation, they may be used interchangeably. However, if the range of the 95 % LoA has significant clinical implications, the two methodologies cannot be used interchangeably [26].

Shirayama et al. [27] used the Galilei dual-Scheimpflug Analyzer, the Humphrey Atlas corneal topographer, the IOLMaster, and a manual keratometer to assess the repeatability and comparability of anterior corneal power values. For the mean keratometry values, each pair of devices recorded a 95 % LoA range of <0.5 D. The corneal measurements from the four devices were found to be highly reproducible and comparable. Another study by Tajbakhsh et al. [28] found that the 95 % LoA range of the keratometry values between the TMS-4 (a Placido disc-based system) and the Orbscan IIz was 0.8 D which implied that they could not be used interchangeably in clinical practice. In the present study, the 95 % LoA range of the mean keratometry values was approximately 1.37 D with a mean difference of 0.52 D. Classic IOL power calculation formulas usually multiply the keratometry reading by approximately 0.9 [3], so this would translate into a mean difference in lens power prediction of 0.47 D, with 95 % of differences falling within 1.23 D of the spherical equivalent refraction. Because most IOLs are currently available in 0.5 D gradations, these discrepancies might lead to errors in IOL power calculations and cause hyperopia after cataract surgery. According to our data, the mean differences in the corneal power mesurements between the Orbscan II topographer and the iTrace aberrometer were 0.50 (Kf) to 0.55 D (Ks), and the range of the 95 % LoA was 1.40 (Ks) to 1.42 D (Kf). Also, the differences were consistent in both age groups.and in both gender groups. As a consequence, the wide limits of agreement of corneal power mesurements between the two devices observed in the current study were beyond clinically acceptable levels in the prediction of the IOL power.

For the WTW distance measurements, previous studies have verified the accuracy and repeatability obtained with the Orbscan II topographer [10, 12]. Although our study did not demonstrate which device measured the WTW distance more accurately, it should be noted that the iTrace aberrometer measurements were smaller than those of the Orbscan II topographer. Martin et al. [29] found that the mean difference in the WTW distance measurements between the Orbscan and IOLMaster was 0.50 mm (with 95 % LoA of 0.01 to − 1.01 mm), this difference could have practical relevance and they suggested the two devices were not interchangeable for WTW assessment in clinical practice. Another study by Salouti et al. [30] reported that a difference ≥0.50 mm for the WTW distance was considered as clinically relevant. The results of this study show that the range of the 95 % LoA was approximately 0.5 mm (0.47 mm for all the subjects, 0.46 mm for males and 0.49 mm for females) with the Orbscan II topographer, versus the iTrace aberrometer. This difference is clinically relevant in determining an accurate lens diameter for implantation of the STAAR Surgical V4 (Version 4) ICL, because this phakic IOL’s diameter should be approximately 0.5 to 1.0 mm larger than the WTW distance measurement in myopic eyes [8]. Moreover, an over- or under-sized ICL may induce unwanted complications, such as IOL rotation or decentration, pigment dispersion, pupillary block glaucoma, and cataract formation [31, 32].

The observed differences in the corneal power and WTW distance measurements between the two devices are unclear, but the use of distinct methodologies in each device might induce this tendency. For the keratometry values, both systems measure anterior corneal curvature using the Placido rings while the Orbscan II topographer can analyze the posterior surface with the help of scanning slits. The accuracy of the WTW distance measurements relies on the quality of the anterior segment images. With the Orbscan II topographer, this is composed of multiple scanning slit images, furthermore, the Orbscan II automatically rejects low-quality images. While with the iTrace aberrometer, the quality of the image is discernible by the operator. On the other hand, a longer capture time to acquire multiple images by the Orbscan II topographer might affect fixation and data accuracy, leading to a poor agreement with the data measured by the iTrace aberrometer.

There are several limitations of the present study. First, the keratometry values were calculated solely from the anterior corneal surface. Second, our comparisons were merely restricted to normal, healthy corneas from a limited age group of refractive surgery and Orthokeratology candidates, the keratometry and WTW distance scan images were of excellent quality. In hyperopic patients or older patients with cataract, the results may be different. Further studies are required to comprehensively assess the agreement of the corneal power and WTW distance measurements obtained by the two devices in such cases. Finally, our study only used one operator for each device, the interobserver repeatability of the corneal power and WTW distance measurements by the two devices deserves further investigations.

## Conclusions

The present study demonstrated that measurements of the corneal power and WTW distance with the Orbscan II topographer were larger than those obtained with the iTrace aberrometer in the assessment of myopic eyes. The differences between the two devices were not within clinically acceptable levels, and therefore the two devices could not be used interchangeably in clinical practice.

### Ethics approval and consent to participate

The ethics committee of Xiangya Hospital approved this study (No. 201408390). Adults and parents of juveniles provided the written informed consent in accordance with the Declaration of Helsinki. The trial registration was requested on August 2nd, 2014.

### Consent for publication

Not applicable for this study.

### Availability of data and materials

All the data supporting the conclusions of this article is included within the article.

## Abbreviations

BCVA: best corrected distance visual acuity
D: diopter
HOAs: higher-order optical aberrations
ICL: implantable collamer lenses
IOL: intraocular lens
Kf: keratometry in the flat meridian
Ks: keratometry in the steep meridian
LoA: limits of agreement
WTW: white-to-white

## References

[1] Qazi MA, Cua IY, Roberts CJ, Pepose JS (2007). Determining corneal power using Orbscan II videokeratography for intraocular lens calculation after excimer laser surgery for myopia. J Cataract Refract Surg.

[2] Martinez CE, Klyce SD (1996). Corneal topography in cataract surgery. Curr Opin Ophthalmol.

[3] Olsen T (2007). Calculation of intraocular lens power: a review. Acta Ophthalmol Scand.

[4] Seitz B, Langenbucher A (2000). Intraocular lens power calculation in eyes after corneal refractive surgery. J Refract Surg.

[5] Rosa N, Capasso L, Lanza M, Borrelli M (2009). Clinical results of a corneal radius correcting factor in calculating intraocular lens power after corneal refractive surgery. J Refract Surg.

[6] Lovisolo CF, Reinstein DZ (2005). Phakic intraocular lenses. Surv Ophthalmol.

[7] Perez-Cambrodi RJ, Pinero DP, Ferrer-Blasco T, Cervino A, Brautaset R (2013). The posterior chamber phakic refractive lens (PRL): a review. Eye (Lond).

[8] Seo JH, Kim MK, Wee WR, Lee JH (2009). Effects of white-to-white diameter and anterior chamber depth on implantable collamer lens vault and visual outcome. J Refract Surg.

[9] Reinstein DZ, Lovisolo CF, Archer TJ, Gobbe M (2013). Comparison of postoperative vault height predictability using white-to-white or sulcus diameter-based sizing for the visian implantable collamer lens. J Refract Surg.

[10] Wang L, Auffarth GU (2002). White-to-white corneal diameter measurements using the eyemetrics program of the Orbscan topography system. Dev Ophthalmol.

[11] Cairns G, McGhee CN (2005). Orbscan computerized topography: attributes, applications, and limitations. J Cataract Refract Surg.

[12] Baumeister M, Terzi E, Ekici Y, Kohnen T (2004). Comparison of manual and automated methods to determine horizontal corneal diameter. J Cataract Refract Surg.

[13] Menassa N, Kaufmann C, Goggin M, Job OM, Bachmann LM, Thiel MA (2008). Comparison and reproducibility of corneal thickness and curvature readings obtained by the Galilei and the Orbscan II analysis systems. J Cataract Refract Surg.

[14] Crawford AZ, Patel DV, McGhee CN (2013). Comparison and repeatability of keratometric and corneal power measurements obtained by Orbscan II, Pentacam, and Galilei corneal tomography systems. Am J Ophthalmol.

[15] Oliveira CM, Ferreira A, Franco S (2012). Wavefront analysis and Zernike polynomial decomposition for evaluation of corneal optical quality. J Cataract Refract Surg.

[16] Maeda N (2009). Clinical applications of wavefront aberrometry - a review. Clin. Experiment. Ophthalmol.

[17] Marcos S. Aberrometry: basic science and clinical applications. Bull Soc Belge Ophtalmol. 2006;197–213. 17265799

[18] Jun I, Choi YJ, Kim EK, Seo KY, Kim TI (2012). Internal spherical aberration by ray tracing-type aberrometry in multifocal pseudophakic eyes. Eye (Lond).

[19] Rozema JJ, Van Dyck DE, Tassignon MJ (2005). Clinical comparison of 6 aberrometers. Part 1: Technical specifications. J Cataract Refract Surg.

[20] Visser N, Berendschot TT, Verbakel F, Tan AN, de Brabander J, Nuijts RM (2011). Evaluation of the comparability and repeatability of four wavefront aberrometers. Invest Ophthalmol Vis Sci.

[21] Bland JM, Altman DG (1986). Statistical methods for assessing agreement between two methods of clinical measurement. Lancet.

[22] Alio JL, Abdelghany AA, Fernandez-Buenaga R (2015). Enhancements after cataract surgery. Curr Opin Ophthalmol.

[23] Gomez ML (2014). Measuring the quality of vision after cataract surgery. Curr Opin Ophthalmol.

[24] Norrby S (2008). Sources of error in intraocular lens power calculation. J Cataract Refract Surg.

[25] Olsen T (1986). On the calculation of power from curvature of the cornea. Br J Ophthalmol.

[26] Bland JM, Altman DG (1999). Measuring agreement in method comparison studies. Stat Methods Med Res.

[27] Shirayama M, Wang L, Weikert MP, Koch DD (2009). Comparison of corneal powers obtained from 4 different devices. Am J Ophthalmol.

[28] Tajbakhsh Z, Salouti R, Nowroozzadeh MH, Aghazadeh-Amiri M, Tabatabaee S, Zamani M (2012). Comparison of keratometry measurements using the Pentacam HR, the Orbscan IIz, and the TMS-4 topographer. Ophthalmic Physiol Opt.

[29] Martin R, Ortiz S, Rio-Cristobal A (2013). White-to-white corneal diameter differences in moderately and highly myopic eyes: partial coherence interferometry versus scanning-slit topography. J Cataract Refract Surg.

[30] Salouti R, Nowroozzadeh MH, Zamani M, Ghoreyshi M, Khodaman AR (2013). Comparison of Horizontal corneal diameter measurements using the Orbscan IIz and Pentacam HR systems. Cornea.

[31] Kohnen T, Kook D, Morral M, Guell JL (2010). Phakic intraocular lenses: part 2: results and complications. J Cataract Refract Surg.

[32] Guell JL, Morral M, Kook D, Kohnen T (2010). Phakic intraocular lenses part 1: historical overview, current models, selection criteria, and surgical techniques. J Cataract Refract Surg.

